# A functional genetic variation of *SLC6A2* repressor hsa-miR-579-3p upregulates sympathetic noradrenergic processes of fear and anxiety

**DOI:** 10.1038/s41398-018-0278-4

**Published:** 2018-10-19

**Authors:** L. G. Hommers, J. Richter, Y. Yang, A. Raab, C. Baumann, K. Lang, M. A. Schiele, H. Weber, A. Wittmann, C. Wolf, G. W. Alpers, V. Arolt, K. Domschke, L. Fehm, T. Fydrich, A. Gerlach, A. T. Gloster, A. O. Hamm, S. Helbig-Lang, T. Kircher, T. Lang, C. A. Pané-Farré, P. Pauli, B. Pfleiderer, A. Reif, M. Romanos, B. Straube, A. Ströhle, H.-U. Wittchen, S. Frantz, G. Ertl, M. J. Lohse, U. Lueken, J. Deckert

**Affiliations:** 1 Interdisciplinary Center for Clinical Research, University Hospital of Würzburg, Würzburg, Germany; 2 Center for Mental Health, Department of Psychiatry, Psychosomatics and Psychotherapy, University Hospital of Würzburg, Würzburg, Germany; 3 Comprehensive Heart Failure Center (CHFC), University Hospital of Würzburg, Würzburg, Germany; 4 Department of Biological and Clinical Psychology/Psychotherapy, University of Greifswald, Greifswald, Germany; 5 Department of Psychiatry and Psychotherapy & Marburg Center for Mind, Brain and Behavior - MCMBB, Phillips-University Marburg, Marburg, Germany; 6 Institute of Pharmacology and Toxicology, University of Würzburg, Würzburg, Germany; 7 Department of Psychiatry and Psychotherapy, Medical Center – University of Freiburg, Faculty of Medicine, University of Freiburg, Freiburg, Germany; 8 Department of Psychiatry and Psychotherapy, Charité University Medicine, Berlin, Germany; 9 Department of Psychology, School of Social Sciences, University of Mannheim, Mannheim, Germany; 10 Department of Psychiatry, University Hospital Münster, Münster, Germany; 11 Department of Psychology, Humboldt-University, Berlin, Germany; 12 Department of Clinical Psychology and Psychotherapy, University of Cologne, Cologne, Germany; 13 Department of Psychology, University of Basel, Basel, Switzerland; 14 Department of Clinical Psychology and Psychotherapy, University of Hamburg, Hamburg, Germany; 15 Institute of Clinical Psychology and Psychotherapy, Technische Universität Dresden, Dresden, Germany; 16 Christoph-Dornier-Foundation for Clinical Psychology, Bremen, Germany; 17 Center of Mental Health, Department of Biological Psychology, Clinical Psychology, and Psychotherapy, Julius-Maximilians-Universität Würzburg, Würzburg, Germany; 18 Department of Clinical Radiology, University Hospital Münster, Münster, Germany; 19 Department of Psychiatry, Psychosomatic Medicine and Psychotherapy, University Hospital Frankfurt, Frankfurt am Main, Germany; 20 Center of Mental Health, Department of Child and Adolescent Psychiatry, Psychosomatics and Psychotherapy, University Hospital of Würzburg, Würzburg, Germany; 21 Clinical Psychology & Psychotherapy RG, Department of Psychiatry & Psychotherapy, Ludwig Maximilans Universität Munich, Munich, Germany; 22 Department of Internal Medicine I, University Hospital of Würzburg, Würzburg, Germany; 23 Max Delbrück Center for Molecular Medicine in the Helmholtz Association, Berlin, Germany

## Abstract

Increased sympathetic noradrenergic signaling is crucially involved in fear and anxiety as defensive states. MicroRNAs regulate dynamic gene expression during synaptic plasticity and genetic variation of microRNAs modulating noradrenaline transporter gene (*SLC6A2*) expression may thus lead to altered central and peripheral processing of fear and anxiety. In silico prediction of microRNA regulation of *SLC6A2* was confirmed by luciferase reporter assays and identified hsa-miR-579-3p as a regulating microRNA. The minor (T)-allele of rs2910931 (MAF_cases_ = 0.431, MAF_controls_ = 0.368) upstream of *MIR579* was associated with panic disorder in patients (*p*_allelic_ = 0.004, *n*_cases_ = 506, *n*_controls_ = 506) and with higher trait anxiety in healthy individuals (*p*_ASI_ = 0.029, *p*_ACQ_ = 0.047, *n* = 3112). Compared to the major (A)-allele, increased promoter activity was observed in luciferase reporter assays in vitro suggesting more effective *MIR579* expression and *SLC6A2* repression in vivo (*p* = 0.041). Healthy individuals carrying at least one (T)-allele showed a brain activation pattern suggesting increased defensive responding and sympathetic noradrenergic activation in midbrain and limbic areas during the extinction of conditioned fear. Panic disorder patients carrying two (T)-alleles showed elevated heart rates in an anxiety-provoking behavioral avoidance test (*F*(2, 270) = 5.47, *p* = 0.005). Fine-tuning of noradrenaline homeostasis by a *MIR579* genetic variation modulated central and peripheral sympathetic noradrenergic activation during fear processing and anxiety. This study opens new perspectives on the role of microRNAs in the etiopathogenesis of anxiety disorders, particularly their cardiovascular symptoms and comorbidities.

## Introduction

Anxiety disorders are a group of frequent mental disorders starting early in life and resulting from complex gene-by-environment interactions^1,2^. Among them, panic disorder (PD) with agoraphobia (AG) is characterized by marked heritability as well as disproportionate generalization of fear to stimuli and contexts not necessarily predictive for actual danger^3–5^. Its clinical syndromes such as panic attacks or anxious apprehension therefore reflect defensive states equivalent to those elicited by actual danger as part of the defensive motivational system as defined by Research Domain Criteria (RDoC)^6^.

The sympathetic noradrenergic system has repeatedly been linked to fear, anxiety, and panic due to its activation in defensive states^7^. Symptoms of autonomic arousal and sympathetic noradrenergic activation such as tachycardia, sweating/increased skin conductance, or trembling are observed during acute threat as well as pathological states of fear, including PD^8,9^. Likewise, symptoms of noradrenergic excess in pheochromocytoma or symptoms elicited by alpha 2-adrenergic receptor antagonist challenge resemble symptoms of sympathetic noradrenergic activation during panic attacks^10,11^ and reports on increased noradrenaline levels during panic attacks may explain these observations^12^.

Central noradrenergic signaling affects multiple processes related to the development of anxiety disorders^13^. Arousal, memory formation, consolidation, and retrieval are involved in defensive responding and fear conditioning^14^. They have been linked to (pre-)motor cortex, medial prefrontal cortex/anterior cingulate cortex, anterior insula, amygdala, hippocampus, and thalamus function^15–17^. Fine-tuning of these neural systems of the defensive motivational system by the noradrenergic system may therefore represent an intermediate phenotype of altered central noradrenergic signaling during fear processing and sympathetic noradrenergic activation.

The noradrenaline transporter SLC6A2 mediates the reuptake of noradrenaline into presynaptic nerve terminals and is therefore a crucial regulator of the noradrenergic system at the synaptic cleft^18^. However, studies focusing on genetic variations spanning *SLC6A2* failed to show a robust and consistent association with trait anxiety or PD so far^19–22^.

In order to bridge the gap between a high heritability but low effect size of single genetic variations, emerging attention has been paid to epigenetic regulation of genes and gene networks^23^. One important, yet understudied mechanism is posttranscriptional repression of gene expression by microRNAs. MicroRNAs are small, non-coding RNA molecules forming incomplete antisense binding to multiple target mRNAs, counteracting expression of multiple genes, and thereby regulating genes on a genetic network level^24,25^. Previous studies have demonstrated the role of microRNAs in synaptic plasticity, memory formation, fear conditioning, behavior as well as mental disorders, including PD and related traits^26–32^.

We hypothesized that genetic variants of microRNAs regulating *SLC6A2* leading to higher microRNA and lower *SLC6A2* expression contribute to pathological fear processing in intermediate phenotypes of sympathetic noradrenergic activation and defensive reactivity. For this purpose, we (1) experimentally analyzed which microRNAs regulated *SLC6A2* expression, (2) studied which single-nucleotide polymorphisms (SNPs) of microRNA genes regulating *SLC6A2* expression were associated with PD and trait anxiety, (3) experimentally analyzed whether the risk genotype modulated expression of its microRNA and whether this microRNA regulated other anxiety candidate genes, and (4) studied whether the risk genotype modulated sympathetic noradrenergic central fear processing and peripheral cardiovascular parameters.

## Materials and methods

### Luciferase reporter and promoter activity assay

Repression of *SLC6A2* expression by microRNAs was analyzed in vitro as previously described^32^. In short, pEZX-MT01 and -MT06 dual firefly/renilla luciferase reporter vectors (GeneCopoeia, Rockville, USA) containing either the 3’-untranslated region (3'UTR) of human *SLC6A2* (HmiT017397-MT01), human *GLRB* (HmiT007719-MT06), human *NPY5R* (HmiT011908-MT06), and human *HTR2B* (HmiT009121-MT06) or no 3’UTR fused to the firefly luciferase (CmiT000001-MT01 or -MT06) were used. Three pmol of small RNA molecules mimicking endogenous microRNAs (mirVana microRNA mimics, Thermo Fisher Scientific, Waltham, USA) were cotransfected along with 40 ng of pEZX-MT01 dual firefly/renilla luciferase reporter vectors containing the 3’UTR of interest in HEK293 cells split into a 96-well plate 4 h prior to transfection with a confluency of about 60%, using the Attractene Transfection Kit (Qiagen, Venlo, Netherlands). Cells were tested for contamination with mycoplasma on a regular basis. Post-transfection, cells were incubated for 40–48 h and luciferase activity was assessed using the LucPair Duo-Luciferase Assay Kit (GeneCopoeia, Rockville, USA) and an EnVision 2104 Multilabel Reader (PerkinElmer, Waltham, MA). Assays were repeated three times in technical triplicates for each microRNA tested in each single experiment. Luciferase activity suppression by each tested microRNA was normalized as follows. (I) Luciferase activity was normalized to the renilla activity of each well and than averaged in technical triplicates (termed relative luciferase activity), thereby normalizing for different transfection efficiencies in different wells. (II) Relative luciferase activity of a microRNA was normalized to the relative luciferase activity of microRNA-untransfected control conditions for the same vector (termed maximal luciferase activity), thereby normalizing for unspecific effects on the cellular level upon cotransfection of a microRNA. (III) Maximal luciferase activity of each microRNA tested on a 3’UTR vector of interest was normalized to the maximal luciferase activity of the same microRNA on a control vector containing no 3’UTR (termed normalized luciferase activity), thereby normalizing for unspecific microRNA binding and effects at other parts of the vector sequence than the 3’UTR of interest.

Regulation of promoter activity by risk genotype of *MIR579* rs2910931 was assessed as previously reported^33^. In brief, single-stranded DNA oligonucleotides containing the different alleles rs2910931 along with a −20/+20 bp upstream/downstream flanking region were tested for modulation of relative luciferase activity using pGL4.23 as the firefly expressing reporter and pGL4.74 as the renilla expressing control (Promega Corporation, Madison, USA). Details of the primer design are given in supplemental methods.

### Prediction and validation of microRNA binding at the 3’UTR of *SLC6A2*

Repression of *SLC6A2* gene expression by microRNAs was predicted in silico using TargetScanHuman 6.2^34^, DIANA microT-CDS^35^, and miRDB^36^. Predicted microRNAs were validated by means of dual firefly/renilla luciferase reporter assays considering previous results^32^ when showing either a score <−0.275 for TargetScan (site-specific context score), >0.75 for Diana microT-CDS, or >70 for mirDB. Of the 43 microRNAs tested (supplemental Table 2), 10 microRNAs showed a repression of normalized luciferase activity to <85% and no overlap of their 95% confidence interval (CI) with that of negative control ath-miR-159a, highly suggesting a functional relevance in vivo. These microRNAs were considered to be candidates for further genetic and functional analysis.

### Samples

Detailed descriptions of all samples have been published^9,16,33,37^. Sample characteristics are summarized in supplementary table 1. Briefly, unrelated German PD patients (*n* = 506, mean age 35.2 ± 10.7 years, 70% female) from the BMBF “Panic-Net” study wave 1 (diagnosis of PD/AG 100%) and wave 2 (diagnosis of PD/AG 87%) were recruited at multiple locations across Germany. Diagnosis was established using the Composite International Diagnostic Interview according to Diagnostic and Statistical Manual of Mental Disorders, Fourth Edition (DSM-IV) criteria^38^. For genetic association analyses, independent controls (*n* = 506, mean age 30.5 ± 8.3, 70% female), screened for absence of Axis I disorders by experienced psychologists on the basis of the Mini International Neuropsychiatric Interview according to the criteria of DSM-IV^39^, were selected from the CRC-TRR-58 MEGA study in order to achieve 1:1 matching for sex and age. Genetic association analyses with dimensional anxiety traits were performed using the Agoraphobic Cognitions Questionnaire (ACQ)^40^ as well as Anxiety Sensitivity Index (ASI)^41^ and the complete CRC-TRR-58 MEGA study sample (*n* = 3112). Individuals with ASI or ACQ scores deviating more than three standard deviations (SDs) from the mean were excluded prior to analysis. For the analysis of functional magnetic resonance imaging (fMRI) in a healthy control sample, genotype information was available in *n* = 40 of *n* = 60 participants. Further details concerning this sample have been published^42^. Data on genotype and the behavioral avoidance test (BAT) were available in *n* = 276 patients PD/AG patients of the BMBF “Panic-Net” study wave 1. Further details concerning this sample have been published^9^.

All participant had given their written informed consent according to the Helsinki guidelines in their updated form. The CRC-TRR-58 MEGA study was approved by the Ethics committee of the Medical Faculty of the Julius-Maximilans-University Würzburg (EK 7/08). The BMBF “PanicNet” RCT project was approved by the Ethics Committees of the Medical Faculty of the Technische Universität Dresden (EK 164082006) and the German Psychological Society (AH11.2009) for wave I and II, respectively. Neuroimaging was approved by the Ethics Committee of the Medical Faculty of the Rheinisch-Westfaelische Technische Hochschule University Aachen (EK 073/07).

### Genotyping

Genomic DNA of all attendees was extracted from venous blood by a routine desalting method. Genotyping of samples for SNPs of microRNA genes regulating *SLC6A2* expression was performed using competitive allele-specific polymerase chain reaction (KASP; LGC Group Ltd., Teddington, UK). All samples were subjected to a stringent quality-control pipeline, accounting for call rates >0.99, Hardy–Weinberg equilibrium (HWE) >0.1, minor allele frequency (MAF) > 0.1, and principal component analysis more than four-fold SD of the first three principal components.

### Analysis on the level of central and peripheral systems of fear processing and anxiety

Acquisition of fMRI data in subjects completing a one-session differential fear conditioning and extinction task has been described previously^42,43^. Briefly, visually presented colored geometrical forms served as conditioned stimulus (CS) during multi-trial familiarization (F), acquisition (A), and extinction (E) phases of the task. White noise adjusted to individual perception of aversiveness served as the unconditioned stimulus (US). The US was pseudorandomly paired with one of the CS during acquisition such that equal proportions of CS+paired and CS+unpaired trials were obtained during acquisition. Only CS+unpaired trials were used for further analyses to avoid confounding between CS+ and US processing. The acquisition and extinction phase were split into halves to assess time-dependent processes. Of *n* = 60 quality-controlled fMRI datasets, genetic information was available for *n* = 40 subjects.

Acquisition of BAT data has been described previously^9^. Briefly, PD/AG patients were asked to stay in a closed and dark test chamber for approximately 10 min, thus inducing agoraphobic anxiety and avoidance behavior. Autonomic (heart rate) and subjective readouts (reported fear) were collected prior, during, and after exposure. Active avoidance (escaping the test chamber) was noted. Patients without AG were excluded owing to the specificity of the test to agoraphobic fear and avoidance. Furthermore, patients with missing subjective and physiological data due to complete avoidance of exposure to the test chamber or technical equipment failures were excluded, resulting in a final sample of 276 patients.

More details are given in supplemental methods.

### Statistical analysis

Statistical analyses using Student’s *t* test were conducted as implemented in Prism 6 (Graph-Pad, La Jolla, USA) or SPSS 24 (IBM, Armonk, USA). Statistical analyses of genotype data were performed using SPSS 24 applying chi-square-tests for deviation from HWE as well as case–control associations of allele or genotype counts and linear regression as well as multivariate analysis of variance (ANOVA) for gene dosage effects on ASI or ACQ. We achieve a power of 63% to detect polymorphisms conveying a relative risk of 1.5 to develop PD assuming a MAF of 0.05^44^. Correction for *n*-multiple tests was conducted using Sidák’s approach and corrected *p* values are given as *p*_c_ = 1− (1 – *p*)^*n*^. All tests were conducted two sided.

Statistical analysis of fMRI data was conducted as described previously^16^. On the group level, contrast images for CS+unpaired and CS− of the first and second halves of the acquisition and extinction phase were entered into a flexible factorial design treating subjects as random variables and the fMRI center as a covariate. Contrasts of interest were the main effect of genotype group during first and second halves of acquisition and extinction, as well as the interaction effect of group × CS (CS + unpaired vs. CS−) for each phase. In case of significant *F* tests, *t* tests were used to localize the direction of effects. Monte-Carlo simulation was adopted to establish a voxel contiguity threshold to correct for multiple comparisons at the level of *p* < 0.05 (family-wise error corrected (FWE))^45^. Assuming an individual voxel type I error of *p* < 0.005, a cluster extent of 142 contiguous resampled voxels allowed to correct for multiple voxel comparisons at *p* < 0.05. As this cluster-based threshold might not detect effects in smaller regions, region-of-interest analyses were performed for the amygdala using the automated anatomic labeling atlas to generate two unilateral masks with small volume correction at *p* < 0.05 FWE-corrected, using a cluster-forming threshold of *p* < 0.001 uncorrected^46^. Beta values from significant clusters were extracted and used for bar graph visualization. Detailed information is provided in supplemental methods.

In order to test avoidance behavior during the BAT, the frequency of active avoidance as well as the duration of tolerated exposure in active escapers were analyzed as a function of genotype using chi-square tests and a model of variance. The genotype effect on reported fear and heart rate response was tested applying a mixed model of variance including genotype and avoidance (active vs. no avoidance) as a between-subject factor and BAT phase (anticipation vs. exposure vs. recovery) as a within-subject factor. All *p* values correspond to two-sided testing.

## Results

### Association analysis with PD and trait anxiety of SNPs of microRNAs regulating *SLC6A2* expression

**Fig. 1:**
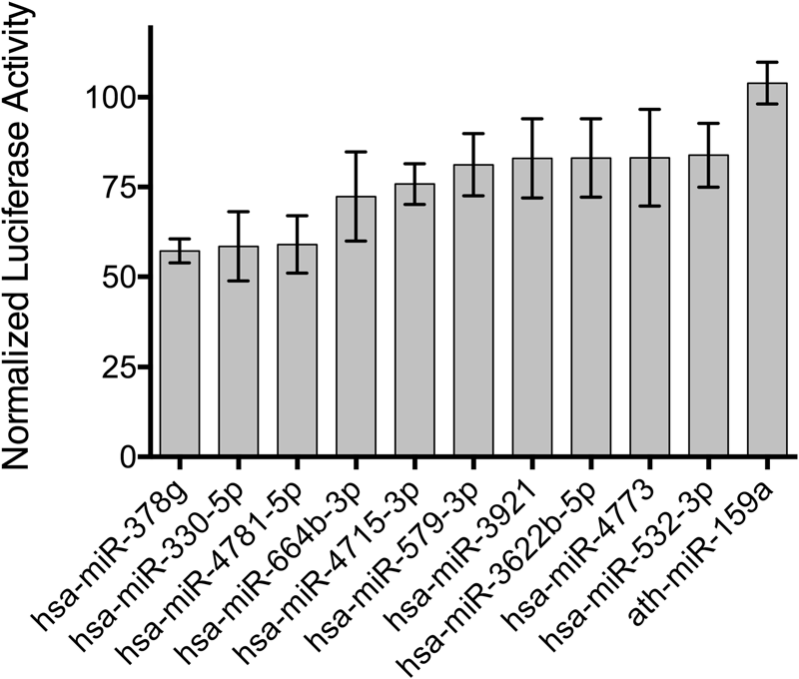
MicroRNA-mediated regulation of *SLC6A2* expression. Data show mean repression of normalized firefly luciferase activity upon cotransfection of a dual luciferase reporter vector containing the 3’UTR of *SLC6A2* with the microRNA as indicated and 95% confidence intervals. Data were normalized to same-well renilla activity as well as to the activity of a no 3’UTR-containing vector (*n* = 5 technical triplicates). The 95% confidence intervals of all microRNAs shown were outside the 95% confidence interval of the negative control microRNA ath-miR-159a (95% CI [98.2, 109.7]) and thus considered to be significantly regulating *SLC6A2*

TagSNPs with a MAF > 0.1 were determined for each of the ten candidate microRNA genes identified by means of luciferase reporter assays (Fig. 1) in a flanking region of 2000 bp upstream and 500 bp downstream^47^. No SNPs were identified for *MIR532*, *MIR664B*, *MIR3921*, and *MIR4781*. For the purpose of completeness, rs7194256 located in the 3’UTR of *SLC6A2* was also included in order to assess modulation of microRNA binding at the 3’UTR of *SLC6A2*. The linkage disequilibrium structure is shown in supplementary figure 1 to 6. Genotyping of rs12151009 tagging *MIR330* failed quality-control criteria and was excluded. Seventeen SNPs were left for further analyses.

Case–control association analysis with PD/AG and MAFs for each SNP are given in Table 1. Genotype frequencies of all SNPs resembled those of European reference samples (HapMap CEU and TSI) as well as the 1000Genomes project (MAF(T) = 0.3624), suggesting no confounding by sample- or population-specific effects. The minor (T)-allele of rs2910931 tagging *MIR579* was nominally associated with PD/AG (*p*_allelic_ = 0.004), as was the major (A)-allele of rs2582372 tagging *MIR3662A* (*p*_allelic_ = 0.023). Upon correction for multiple testing, the allele count of rs2910931 still showed a trend toward significance (*p*_c_ = 0.069). Association of rs2910931 with dimensional trait anxiety as analyzed by linear regression adjusted for sex and age showed a significant association between the number of minor (T)-alleles carried and ASI (beta = 0.371, *p* = 0.029, 95% CI [0.039, 0.702]) as well as ACQ scores (beta = 0.012, *p* = 0.041, 95% CI [0.000, 0.023]), in line with an additive genetic model. Multiway ANOVA suggested no interaction with sex or dichotomized age.

**Table 1:** Genetic association analysis in a case–control sample

SNP	Gene	MAF		*p*(additive)	*p*(allelic)
		Cases	Controls		
rs1333953	*MIR378G*	0.356	0.353	0.887	0.896
rs1760512	*MIR378G*	0.395	0.400	0.478	0.832
rs10874892	*MIR378G*	0.239	0.237	0.836	0.901
rs11165236	*MIR378G*	0.221	0.240	0.492	0.299
rs4847356	*MIR378G*	0.233	0.231	0.490	0.902
rs2286755	*MIR330*	0.162	0.164	0.952	0.919
rs7252448	*MIR330*	0.253	0.253	0.443	0.981
rs12050652	*MIR4715*	0.422	0.463	0.111	0.065
rs2910931	*MIR579*	0.431	0.368	0.018*	0.004*
rs66683138	*MIR3622A/B*	0.252	0.226	0.330	0.175
rs522881	*MIR3622A/B*	0.441	0.423	0.517	0.420
rs554687	*MIR3622A/B*	0.430	0.438	0.440	0.701
rs2582372	*MIR3622A/B*	0.146	0.183	0.024*	0.023*
rs17384485	*MIR3622A/B*	0.361	0.348	0.616	0.531
rs10432476	*MIR4773*	0.453	0.452	0.566	0.995
rs1046668	*MIR4773*	0.173	0.163	0.829	0.550
rs7194256	*SLC6A2*	0.139	0.139	0.658	0.382

Single-nucleotide polymorphisms flanking genes as indicated were genotyped. Association with the diagnosis of panic disorder was calculated for an additive as well as an allelic genetic model. Nominally significant associations are highlighted in bold letters. Upon correction for multiple testing, association of rs2910931 with panic disorder (*p*_c(allelic)_ = 0.069) remained at a trend toward significance

### Regulation of MIR579 promoter activity by rs2910931 and anxiety candidate gene expression by hsa-miR-579-3p

**Fig. 2:**
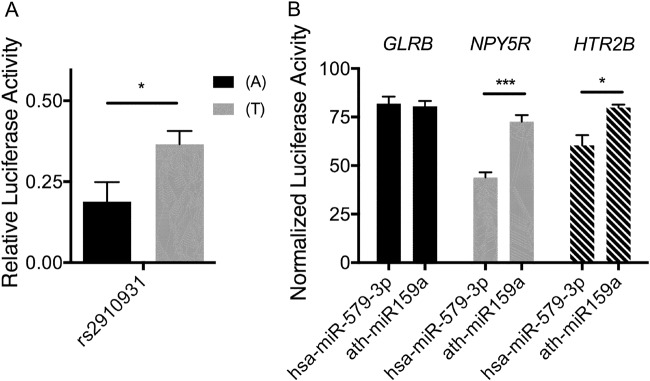
Dual luciferase reporter assay for modulation of *MIR579* expression by upstream genetic variation and hsa-miR-579-3p-mediated regulation of anxiety candidate gene expression. **a** Data show mean relative firefly luciferase activity under a minimal promoter sequence containing the (A)- and (T)-allele of rs2910931 upstream of *MIR579* as indicated. Data were normalized to same-well renilla activity and are shown as mean and standard error (*n* = 7 technical triplicates, *p < 0.05 using a two-sided *t* test). **b** Data show mean repression of normalized firefly luciferase upon cotransfection with hsa-miR-579-3p and ath-miR159a (negative control) as indicated. Dual luciferase reporter assays contained the 3’UTR of the corresponding receptor genes as indicated. Data were normalized to negative-control-3’UTR relative firefly activity for each microRNA as indicated (*n* = 5 technical triplicates, *p < 0.05, ***p < 0.005)

Rs2910931 is located within the putative promoter region 2 kB upstream of *MIR579*. Assessing promoter activity in vitro by means of luciferase reporter assays revealed a significantly higher relative luciferase activity for the minor (T)-allele compared to the major (A)-allele (Fig. 2a, *p* = 0.041). This result suggests a higher expression of *MIR579* and hsa-miR-579-3p as well as decreased expression of *SLC6A2* in vivo.

Using TargetScan 6.2 and Diana microT-CDS, in silico prediction yielded high scores for 3’UTR binding of hsa-miR-579-3p for *GLRB*, *HTR2B*, and *NPY5R*, in line with regulation of possible anxiety candidate genes^33,48,49^. A significant repression of luciferase activity upon cotransfection of hsa-miR-579-3p was observed for vectors containing the 3’UTR of *HTR2B* and *NPY5R* (Fig. 2b).

### Effects of rs2910931 on neural substrates of fear conditioning and extinction in healthy controls

**Fig. 3:**
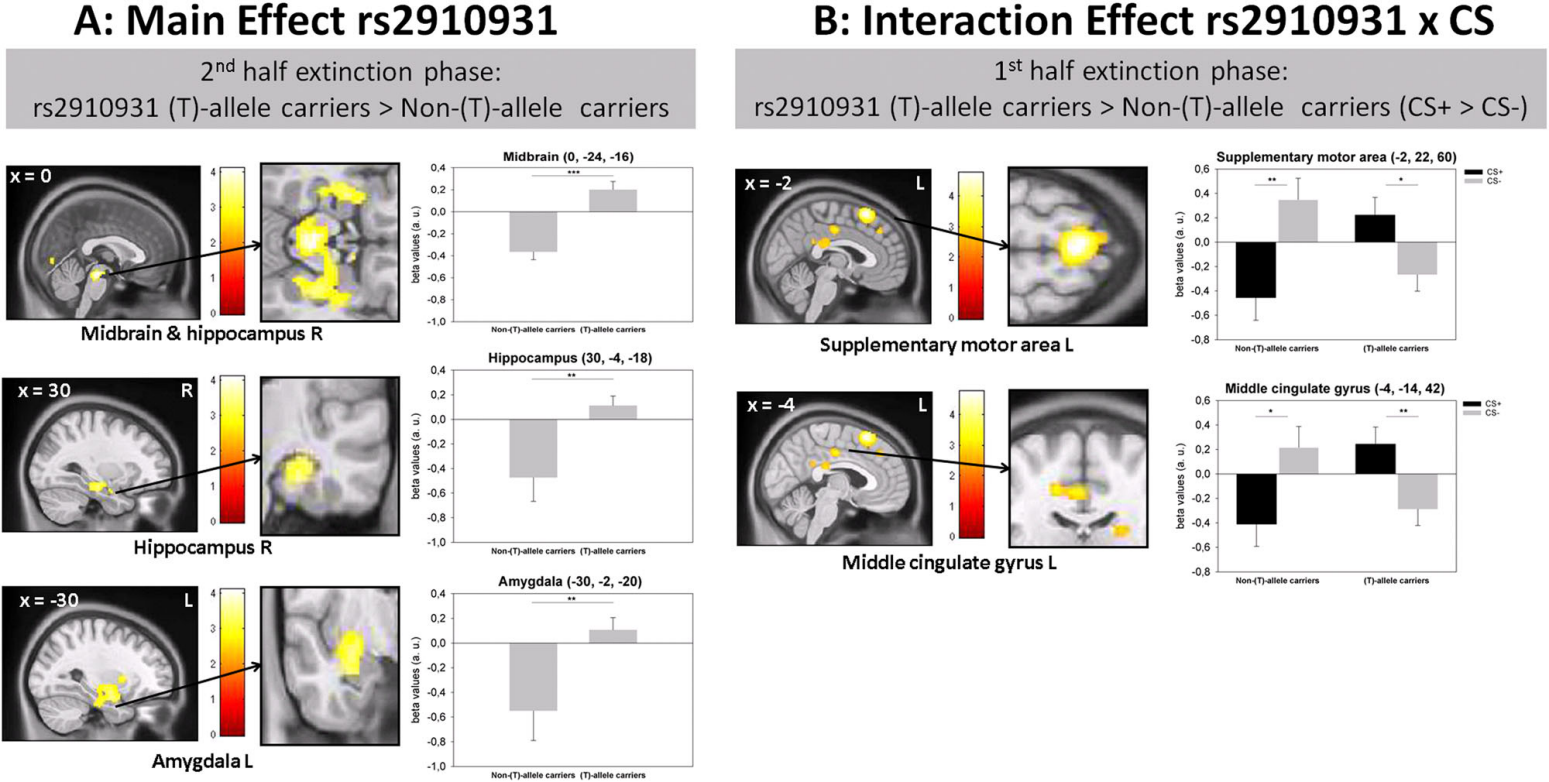
Neurofunctional activation patterns of fear-related brain structures in healthy controls during fear conditioning and extinction. Healthy controls (*n* = 40) were subjected to an experimental fear conditioning and extinction task. Activation of brain regions was analyzed by means of fMRI as a function of minor (T)-alleles of rs2910931 (TT+AT: *n* = 25; AA: *n* = 15). Beta values from significant clusters were extracted and used for bar graph visualization (a.u.). **a** Activation of midbrain/periaqueductal gray, amygdala, and hippocampus regardless of conditioned stimulus presented during the second half of the extinction phase as a function of rs2910931 genotype group. **b** Interaction of rs2910931 genotype group with the conditioned stimulus presented during the first half of the extinction phase. *p < 0.05, **p < 0.01, and ***p < 0.001. CS+ conditioned stimulus that was followed by the unconditioned stimulus (US); CS− conditioned stimulus that was never followed by the US

Neural activation during fear conditioning and extinction was assessed by means of fMRI^42^. Genetic information was available in *n* = 40 of *n* = 60 participants. Owing to the limited sample size, carriers of at least one minor (T)-allele were considered to be at higher risk for anxiety-related measures (*n* = 25), leaving (AA)-carriers in the low-risk group (*n* = 15). Activation patterns were significantly modulated by rs2910931 as summarized in Table 2. A genotype main effect was observed in the late extinction phase. Post hoc *t* tests showed that subjects carrying at least one minor (T)-allele exhibited enhanced neural activation in brain regions related to sympathetic noradrenergic activation and fear processing such as the midbrain/periaqueductal gray (PAG), amygdala, and hippocampus, regardless of the CS presented. When carrying no minor (T)-allele, a pronounced deactivation was noted in these regions (Fig. 3a). Moreover, an interaction between genotype and CS presented was observed during the first half of the extinction phase. Minor (T)-allele carriers showed enhanced neural activation of higher-order cortical regions such as the supplementary motor area (SMA) and the middle cingulate gyrus upon presentation of the CS+, while those not carrying any (T)-allele showed enhanced neural activation upon presentation of the CS− (Fig. 3b).

**Table 2:** Genetic imaging analysis of rs2910931

Contrast/region	Side	Voxels	*x*	*y*	*z*	*F* or *t*	*p* ^a^
Main group effects							
Acquisition first phase						No differential activation	
Acquisition second phase							
Inferior occipital lobe	L	156	−12	−96	−10	17.12	<0.001
Post hoc *t* tests							
Acquisition second phase: risk>no risk						No differential activation	
Acquisition second phase: no risk>risk							
Inferior occipital lobe	L	225	−12	−96	−10	4.14	<0.001
Extinction first phase						No differential activation	
Extinction second phase							
Midbrain (incl. right hippocampus)		550	0	−24	−16	16.85	<0.001
Inferior parietal lobe	L	159	−54	−42	54	9.62	0.002
Hippocampus (2 mm dev.)	L	355	30	−4	−18	12.33	0.001
Amygdala^b^	L	16	−30	−2	−20	12.04	0.013
Post hoc *t* tests							
Extinction second phase: risk>no risk							
Midbrain (incl. right hippocampus)		1018	0	−24	−16	4.11	<0.001
Inferior parietal lobe	L	272	−54	−42	54	3.10	0.001
Hippocampus (2 mm dev.)	L	737	30	−4	−18	3.51	<0.001
Amygdala^b^	L	36	−30	−2	−20	3.47	0.007
Lingual gyrus	L	196	−4	−78	−4	3.15	0.001
Extinction second phase: no risk>risk						No differential activation	
Group × CS interaction effects							
Acquisition first phase							
Inferior frontal gyrus pars triangularis	L	235	−44	28	26	13.11	<0.001
Post hoc *t* tests							
Acquisition first phase: risk>no risk (CS + unpaired > CS−)						No differential activation	
Acquisition first phase: no risk>risk (CS + unpaired > CS−)							
Inferior frontal gyrus pars triangularis	L	429	−44	28	26	3.62	<0.001
Acquisition second phase							
Caudate nucleus	L	378	−8	14	12	13.17	<0.001
Middle temporal gyrus (4.47 mm dev.)	R	192	36	−60	4	12.50	<0.001
Post hoc *t* tests							
Acquisition second phase: risk>no risk (CS + unpaired > CS−)							
Caudate nucleus	L	564	−8	14	12	4.31	<0.001
Middle temporal gyrus (4.47 mm dev.)	R	348	36	−60	4	3.55	<0.001
Acquisition second phase: no risk > risk (CS + unpaired > CS−)						No differential activation	
Extinction first phase							
Supplementary motor area	L	735	−2	22	60	22.76	<0.001
Caudate nucleus (4.90 mm dev.)	R	219	20	12	28	12.92	<0.001
Middle frontal gyrus (3.46 mm dev.)	L	218	−26	16	32	12.65	<0.001
Post hoc *t* tests							
Extinction first phase: risk>no risk (CS +> CS−)							
Supplementary motor area	L	1599	−2	22	60	4.77	<0.001
Middle cingulate gyrus	L	543	−4	−14	42	3.30	0.001
Caudate nucleus (4.90 mm dev.)	R	432	20	12	28	3.59	<0.001
Extinction first phase: no-risk > risk (CS +> CS−)	No differential activation						
Extinction second phase	No differential activation						

Main genotype effect of rs2910931 and genotype × CS interaction effects on brain activation patterns during fear acquisition and extinction are given as cluster peak voxels. Risk group status was defined as carrying at least one minor (T)-allele. During the acquisition, only CS+ trials that were not paired with the US were used for analyses

*L* left, *R* right, voxel number of voxels per cluster, *x*, *y*, *z* MNI coordinates, *dev* deviation (in mm) from the identified anatomical structure using anatomic automatic labeling (aal). *CS+* conditioned stimulus followed by the unconditioned stimulus (US) in 50% of trials during acquisition, *CS−* conditioned stimulus never followed by the US

^a^Uncorrected *p* values of whole-brain results with a minimum cluster size of 142 contiguous voxels are given, indicating *p* < 0.05 upon correction for multiple comparisons

^b^Small volume correction using aal masks (FWE correction at *p* < 0.05) with a cluster forming threshold of *p* < 0.001 was applied

### Functional characterization of rs2910931 effects on fear reactivity during the BAT in PD patients

**Fig. 4:**
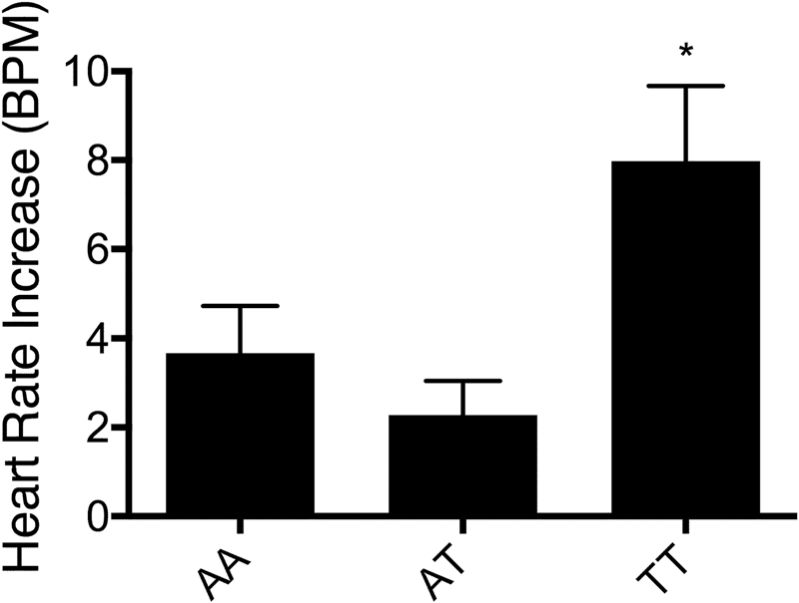
Increase of heart rate in PD patients during the behavioral avoidance test. Patients with panic disorder and agoraphobia (*n* = 276: *n*(AA) = 95, *n*(AT) = 129, *n*(TT) = 52) were subjected to experimental panic exposition using the behavioral avoidance test. Data show the mean difference with standard error of heart rates between the last minute of the anticipation phase and the first minute of the exposure phase during the behavioral avoidance test as a function of rs2910931 genotype. *p < 0.05

To further corroborate the role of rs2910931 on sympathetic noradrenergic activation, PD patients subjected to the BAT provoking anxious apprehension were evaluated. Homozygotic carriers of (T)-alleles showed a significantly higher heart rate increase from the last minute of the anticipation to the first minute of the exposure phase (Fig. 4; *F*(2, 270) = 5.47, *p* = 0.005), irrespective of active avoidance behavior (genotype × behavior *F*(2, 270) = 0.32, *p* = 0.73). For AA-carriers, the heart rate increased from 76.70 ± 1.21 min^−^^1^ prior to exposure to 80.37 ± 1.63 min^−1^ upon exposure. AT-carriers showed an increase from 75.87 ± 0.99 to 78.15 ± 1.32 min^−1^ and TT-carriers from 74.10 ± 1.27 to 82.09 ± 2.01 min^−1^. During the anticipation and recovery phase, heart rates did not differ between genotype and avoidance groups. Overall active avoidance was significantly associated with higher heart rate reactivity (*F*(1, 270) = 30.01, *p* < 0.001), in line with previous findings.

## Discussion

Regulation of gene expression by microRNAs is an important mechanism in neuronal plasticity. Indeed, the role of microRNAs in mental disorders has become an emerging field of research^29,30^. MicroRNAs act on gene networks, making candidate gene-based evaluation of microRNAs a promising approach to identify network regulators of high pathogenic relevance. MicroRNA regulation of *SLC6A2* expression was probed for several reasons. *SLC6A2* is a main regulator of noradrenaline homeostasis by rapidly removing it from the synaptic cleft and the noradrenergic system has been linked with mechanisms of defensive states involved in fear processing and general arousal.

Here we present evidence for the impact of genetic variation of microRNA regulation of *SLC6A2* on the RDoC defensive motivational system in fear and anxiety. Our results suggest that (I) the minor (T)-allele of rs2910931 upstream of *MIR579* leads to increased expression of hsa-miR-579-3p and more effective repression of *SLC6A2* expression along with higher synaptic noradrenaline levels in vivo resulting in (II) higher trait anxiety in healthy individuals possibly due to increased activation of midbrain and limbic areas during fear processing and (III) association with PD mediated by increased sympathetic noradrenergic arousal when entering contexts of potential threat.

Our data suggest that dysbalancing fine-tuning of synaptic noradrenaline homeostasis interferes with successfully establishing fear-inhibitory memory traces and top–down inhibition of defensive responses mediated by brain areas (midbrain/PAG, amygdala) related to sympathetic noradrenergic activation during safety learning^17,50,51^. Moreover, higher-risk (T)-allele carriers showed enhanced recall of the conditioned fear memory during the first half of extinction training as indicated by stronger activation of brain areas like the SMA, commonly associated with fear expression^15^. Taken together, both processes result in pathological arousal and may ultimately contribute to anxiety disorders, sharing increased sympathetic noradrenergic signaling as a common mechanism. This hypothesis was corroborated for PD patients by showing increased sympathetic noradrenergic arousal when undergoing the BAT. Homozygous (T)-allele carriers showed a significantly higher heart rate increase upon agoraphobic fear-provoking exposure to the test chamber. This result is in accordance with a pronounced noradrenaline-mediated autonomic fear response, as supported by previous studies^10,11,13^.

Several limitations apply to our study. While circulating levels of hsa-miR-579 were related to bevacizumab-induced cardiotoxicity^52^, expression of *MIR579* has not yet been described in the human brain^53^ and no neuronal function has been defined so far. Expression quantitative trait loci have been reported for rs2910931 in skin and thyroid tissue (data obtained from the GTEx portal on 24 July 2018), but not for brain tissue or for *MIR579*^54^ as well as no corresponding functional annotation^55^. However, expression in tissues subject to noradrenergic regulation such as the adrenal gland was reported as well as a high probability of transcription start site modulation in the heart of the region flanking *MIR579*, thereby further linking *MIR579* to noradrenergic signaling^55^. *MIR579* is intronic in the zink finger RNA-binding protein *ZFR* and polyadenylation was suggested to modulate a negative feedback loop between both^56^, possibly also relevant at the synapse. We provide additional evidence for a regulatory role of hsa-miR-579-3p at the noradrenergic as well as the serotonergic synapse, by showing regulation of the neuropeptide Y receptor 5 (*NPYR5*) with neuropeptide Y being a co-transmitter of noradrenaline, and the serotonin receptor 2B (*5HTR2B*). Our data thus give a first glimpse into molecular mechanisms by which *MIR579* may act on endophenotypes as well as clinical phenotypes of fear and anxiety, e.g., by regulating not only *SLC6A2* but also other genes involved in fear and anxiety. Future studies adopting next-generation sequencing may give more detailed insight into the relevance of genetic variations at *MIR579* and its flanking region as well as the gene network regulated by it. They may also address the exact mechanisms of hsa-miR-579-3p binding at the mRNA in more detail by using binding-site-directed nucleotide mutagenesis, thereby additionally allowing to assess the role of rare genetic variants at these nucleotides. Conservation of *MIR579* has only been confirmed among *Homo sapiens*, *Pan troglodytes*, *Pongo pygmaeus*, and *Macaca mulatta* and its seed sequence in *SLC6A2* is only incompletely conserved in more distantly related species, such as mice or rat. Validation of *MIR579* functions in mice or rats is therefore not possible. Studies applying cross-linked immunoprecipitation and next-generation sequencing as well as lentivirus-mediated gene overexpression and silencing will allow to identify the gene network regulated by *MIR579*. Further studies may specifically assess the functional role of rs2910931 by simultaneous quantification of *MIR579* and target gene as well as protein expression levels in postmortem brain tissue, neuronal stem cells, or human heterologous expression systems. Although genetic association of rs2910931 with the categorical phenotype PD kept a trend toward significance upon correction for multiple testing and was supported by the association with dimensional anxiety traits, these results need to be considered exploratory. Limited power of the sample in light of the small effect sizes of single genetic variations may account for this, making replication of study results in larger samples a prerequisite to draw final conclusions. Nevertheless, the consistent association with functional central and peripheral endophenotypes of defensive states such as fear conditioning and extinction as well as heart rate support a role in the pathophysiology of fear and anxiety.

Our observations complement a recent report that rs7194256 within the 3’UTR of *SLC6A2* regulating binding of hsa-miR-19a-3p was associated with elevated anxiety measures, arterial noradrenaline levels, depression scores, larger left ventricular mass index, heart rate, as well as higher systolic and diastolic blood pressure levels in healthy controls and patients with affective (depression and PD) and cardiovascular disease^57^. While we failed to observe an association of rs7194256 with trait anxiety or PD, our results share the same direction of effects concerning PD, trait anxiety, and sympathetic noradrenergic endophenotypes for rs2910931 and hsa-miR-579-3p. They add to the observations of Marques and co-workers^57^ by providing evidence for parallel effects of microRNA regulation of *SLC6A2* on central and peripheral sympathetic noradrenergic endophenotypes.

In conclusion, microRNA regulation of *SLC6A2* may contribute to the pathophysiology of fear and anxiety not necessarily by increased basic pathological anxiety but rather by modulating reactions of fear and flight. These reactions are related to increased sympathetic noradrenergic activation, which may represent the common link between anxiety disorders and their cardiovascular symptoms as well as comorbid cardiovascular disease^57–61^. Future studies addressing mechanisms of hsa-miR-579-3p action and its role in clinical cohorts of anxiety and cardiovascular disorders including therapy studies have to further corroborate these hypotheses. As such, studies on microRNAs open up new perspectives on common mechanisms of comorbid disorders and novel therapeutic approaches.

## Electronic supplementary material

Supplemental Material
