# Supplementary material for: A functional genetic variation of SLC6A2 repressor hsa-miR-579-3p upregulates sympathetic noradrenergic processes of fear and anxiety
Source: Transl Psychiatry. 2018 Oct 19;8:226. doi: 10.1038/s41398-018-0278-4 (PMC6195525; doi:10.1038/s41398-018-0278-4)
Supplement: Supplementary file 1 — Supplemental Material [file 41398_2018_278_MOESM1_ESM.docx]

**Supplemental Figure 1:** **LD structure of MIR330.**

**
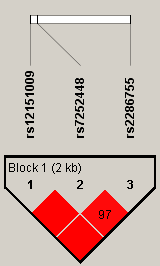
**

**Supplemental Figure 2:** **LD structure of MIR378G.**


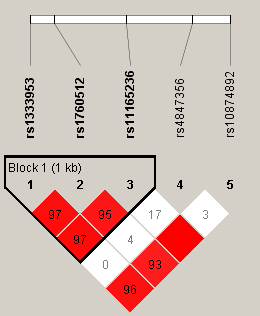


**Supplemental Figure 3:** **LD structure of MIR579.**


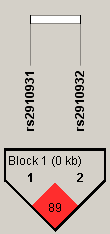


**Supplemental Figure 4:** **LD structure of MIR3622A and MIR3622B.**


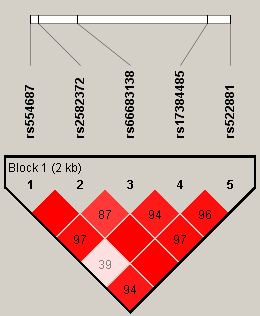


**Supplemental Figure 5:** **LD structure of MIR4715.**


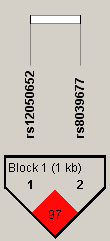


**Supplemental Figure 6:** **LD structure of MIR4773.**


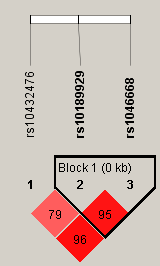


**Supplemental Table 1:** **Description of study samples.** Demographic and clinical characteristics are given for all samples used in the study. The healthy-control sample used for fMRI analysis was stratified for rs2910931 genotype group, with high-risk individuals defined as those carrying at least one minor (T)-allele (AT and TT). Data are given as mean and SD. ASI: Anxiety Sensitivity Index; ACQ: Agoraphobic Cognitions Questionnaire; BDI II: Beck Depression Inventory II; US aversiveness: Unconditioned Stimulus aversiveness ratings in the experimental fear conditioning paradigm applied (10-point Likert Scale).

| Sample | Panic-Net (cases) | | | MEGA study (controls) | | | Panic-Net fMRI (controls) | |
| --- | --- | --- | --- | --- | --- | --- | --- | --- |
|  | *Wave 1* | *Wave 2* | Complete | *Wave 1* | *Wave 2* | Complete | (T)-allele carriers | Non-(T)-allele carriers |
| n | *292* | *214* | 506 | *1628* | *1416* | 3112 | 25 | 15 |
| Female gender | *74.0* | *62.5* | 70.4 | *52.6* | *57.7* | 55.3 | 64.0 | 53.3 |
| Age | *36.0 (10.8)* | *34.0 (10.4)* | 35.2 (10.7) | *25.0 (5.6)* | *25.6 (5.7)* | 25.3 (5.7) | 35.8 (11.2) | 38.3 (10.1) |
| ASI total | *31.1 (11.4)* | *31.0 (10.7)* | 31.1 (11.1) | *13.2 (6.5)* | *14.3 (7.3)* | 13.7 (6.9) | 8.6 (6.9) | 8.7 (9.4) |
| ACQ total | *2.15 (0.57)* | *2.23 (0.56)* | 2.18 (0.57) | *1.35 (0.26)* | *1.34 (0.25)* | 1.35 (0.26) | n.a. | n.a. |
| BDI II total | *n.a.* | *n.a.* | n.a. | *n.a.* | *n.a.* | n.a. | 1.48 (1.71) | 1.40 (2.32) |
| US aversiveness | *n.a.* | *n.a.* | n.a. | *n.a.* | *n.a.* | n.a. | 8.0 (1.2) | 8.4 (0.7) |

**Supplemental Table 2:** **Summary of *SCL6A2* regulation by microRNA.** 44 microRNA selected according to *in silico* prediction for regulation of *SLC6A2* expression by TargetScan 6.2, DIANA-microT-CDS and mirDB were experimentally tested by means of luciferase activity assays. Activity of firefly luciferase upon cotransfection of the corresponding microRNA and normalization to control renilla luciferase activity, negative-control transfection and negative control vector indicates expression regulation of *SLC6A2* by microRNA binding to the 3’UTR of *SLC6A2*. Data are given as mean and SEM of at least n = 3 independent technical triplicates.

| **microRNA** | **mean norm. firefly activity** | **SEM** | **95% CI** |
| --- | --- | --- | --- |
| hsa-miR-129-5p | 1.027 | 0.027 | 0.683 – 1.371 |
| **hsa-miR-1303** | **0.949** | **0.007** | **0.920 – 0.978** |
| hsa-miR-146a-3p | 0.997 | 0.092 | 0.703 – 1.290 |
| hsa-miR-198 | 1.075 | 0.069 | 0.854 – 1.295 |
| hsa-miR-3155b | 1.039 | 0.063 | 0.863 – 1.215 |
| hsa-miR-3200-5p | 0.976 | 0.038 | 0.853 – 1.098 |
| **hsa-miR-330-5p** | **0.585** | **0.039** | **0.488 – 0.681** |
| hsa-miR-361-5p | 1,016 | 0.085 | 0.650 – 1.383 |
| hsa-miR-3616-3p | 0.889 | 0.093 | 0.594 – 1.185 |
| hsa-miR-3619-5p | 1.029 | 0.106 | 0.691 – 1.368 |
| **hsa-miR-3622b-5p** | **0.831** | **0.047** | **0.722 – 0.940** |
| hsa-miR-362-3p | 1.029 | 0.051 | 0.866 – 1.193 |
| hsa-miR-3661 | 0.996 | 0.081 | 0.738 – 1.254 |
| hsa-miR-3665 | 0.991 | 0.062 | 0.793 – 1.188 |
| hsa-miR-3689a-3p | 1.047 | 0.080 | 0.824 – 1.270 |
| **hsa-miR-378g** | **0.572** | **0.015** | **0.539 – 0.606** |
| **hsa-miR-3921** | **0.830** | **0.035** | **0.720 – 0.940** |
| hsa-miR-3925-5p | 1.154 | 0.037 | 0.995 – 1.313 |
| hsa-miR-3943 | 0.833 | 0.066 | 0.624 – 1.042 |
| hsa-miR-4268 | 0.917 | 0.087 | 0.639 – 1.195 |
| hsa-miR-4290 | 0.983 | 0.099 | 0.668 – 1.298 |
| hsa-miR-4450 | 0.976 | 0.049 | 0.821 – 1.131 |
| hsa-miR-4471 | 0.949 | 0.043 | 0.811 – 1.088 |
| hsa-miR-4653-5p | 0.913 | 0.087 | 0.636 – 1.190 |
| hsa-miR-4668-5p | 0.815 | 0.063 | 0.614 – 1.016 |
| hsa-miR-4706 | 0.942 | 0.045 | 0.797 – 1.087 |
| hsa-miR-4713-3p | 0.896 | 0.065 | 0.690 – 1.102 |
| **hsa-miR-4715-3p** | **0.758** | **0.025** | **0.701 – 0.815** |
| hsa-miR-4728-5p | 0.973 | 0.093 | 0.677 – 1.270 |
| hsa-miR-4733-5p | 0.921 | 0.043 | 0.738 – 1.105 |
| hsa-miR-4749-5p | 0.858 | 0.062 | 0.662 – 1.054 |
| **hsa-miR-4773** | **0.832** | **0.057** | **0.697 – 0.966** |
| hsa-miR-4778-5p | 1.013 | 0.056 | 0.835 – 1.191 |
| **hsa-miR-4781-5p** | **0.590** | **0.034** | **0.510 – 0.670** |
| hsa-miR-4786-3p | 0.989 | 0.090 | 0.702 – 1.276 |
| **hsa-miR-532-3p** | **0.839** | **0.036** | **0.750 – 0.928** |
| hsa-miR-548p | 0.940 | 0.046 | 0.793 – 1.087 |
| **hsa-miR-579-3p** | **0.812** | **0.037** | **0.726 – 0.899** |
| hsa-miR-600 | 1,002 | 0.108 | 0.659 – 1.345 |
| hsa-miR-631 | 0.852 | 0.067 | 0.638 – 1.067 |
| **hsa-miR-664b-3p** | **0.724** | **0.039** | **0.599 – 0.848** |
| hsa-miR-718 | 0.995 | 0.049 | 0.838 – 1.152 |
| hsa-miR-761 | 1.032 | 0.037 | 0.913 – 1.151 |
| ***ath-miR-159a*** | ***1.040*** | ***0.027*** | ***0.982 – 1.097*** |
|  |  |  |  |

**Supplementary Table 3.** **Estimation of variance explained by rs2910931**. Proportions of variance explained by rs2910931 in the case-control, ASI and ACQ sample were estimated with the GCTA software (http://cnsgenomics.com/software/gcta.html), using the Restricted Maximum Likelihood procedure. V_(G)_ indicates estimates of genotypic variance, V_p_ indicates estimates of phenotypic variance, V_(G)_/V_p_ is the proportion of phenotypic variance explained by the significant SNP, logL indicates the log-likelihood under the null hypothesis, logL0 the log-likelihood under the alternative hypothesis, LRT indicates the log-likelihood ratio test statistic.

|  | V_(G)_ (s.e.) | V_p_ (s.e.) | V_(G)_/V_p_ (s.e.) | logL | logL0 | LRT | P-value |
| --- | --- | --- | --- | --- | --- | --- | --- |
| Case-control sample (N=1012) | | | | | | | |
| rs2910931 | 0.0019 (0.003) | 0.2502 (0.012) | 0.0075 (0.012) | 191.61 | 188.85 | 5.52 | 0.0094 |
| Control sample ASI (N=3112) | | | | | | | |
| rs2910931 | 0.0687 (0.117) | 41.5373 (1.084) | 0.0017 (0.003) | -7000.16 | -7001.76 | 3.19 | 0.0370 |
| Control sample ACQ (N=3112) | | | | | | | |
| rs2910931 | 0.0001 (0.000) | 0.0516 (0.001) | 0.0014 (0.002) | 2904.45 | 2903.14 | 2.6 | 0.0521 |

**Supplemental Methods**

*Promoter Activity Assay*

Phosphorylated, single-stranded DNA oligonucleotides, containing rs2910931 along with the -20/+20bp up-/downstream region and two restriction sites for 5’ XhoI and 3’ HindIII to allow subcloning into the multiple cloning site of pGL4.23 (Promega Corporation, Madison, WI, USA) were synthesized for each allele as given in the table below (MWG-Biotech AG, Ebersberg, Germany). PGL4.23 allows expression of firefly luciferase by a minimal promoter containing the promotor sequence flanking both SNPs. HEK293 cells were split into a 96-well plate 4h prior to transfection and cotransfected with 20ng pGL4.23 and 20ng of pGL4.74 (Promega Corporation, Madison, WI, USA) using the Attractene Transfection Reagent (Qiagen, Hilden, Germany) and incubated for 40-48h. Luciferase activity was again assessed using the LucPair Luciferase Assay Kit 2.0 (Genecopeia, Rockville, MD, USA) and an EnVision 2104 Multilabel Reader (Perkin Elmer, Waltham, MA, USA). Firefly activity was normalized to renilla activity for each well in technical triplicates. For comparison of allele-specific luciferase activity, a two-sided t-test was conducted.

| rs2910931(A)  reverse complement | 5’-TCGAGTTTGATAGATTCTACATGTT-A-ATAATGTCCATAAAGAAACTA-3’  5’-AGCTTAGTTTCTTTATGGACATTAT-T-AACATGTAGAATCTATCAAAC-3’ |
| --- | --- |
| rs2910931(T)  reverse complement | 5’-TCGAGTTTGATAGATTCTACATGTT-T-ATAATGTCCATAAAGAAACTA-3’  5’-AGCTTAGTTTCTTTATGGACATTAT-A-AACATGTAGAATCTATCAAAC-3’ |

*fMRI data acquisition and analysis*

A differential fear conditioning and extinction task was applied as described previously.^16^ During each trial of the three task-phases (familiarization (F) with 16 trials; acquisition (A) with 32 trials and extinction (E) with 16 trials), each conditioned stimulus (CS, colored geometrical forms) was presented for 2000 ms with a variable inter-trial interval (ITI) of 4.785 to 7.250 sec. An aversive acoustic signal (white noise) presented for 100 ms at 70-105 dB (adjusted to individual perception of aversiveness) served as the unconditioned stiulus (US). Stimuli were presented on MR-compatible LCD goggles or back projection systems and standard headphones using Presentation 11 (Neurobehavioral Systems, Berkeley, USA). During acquisition, the US was pseudorandomly paired with one of the CSs (counterbalanced between subjects; partial reinforcement rate of 50) such that equal proportions of CS+ paired and CS+ unpaired trials were obtained. After each phase of the task, subjective valence and arousal ratings for both CSs were obtained using a five-point Likert Scale (for valence: 1 = ‘very unpleasant’ to 5 = ‘very pleasant’ and for arousal: 1 = ‘not arousing’ to 5= ‘very arousing’). Ratings of one subject were missing due to technical failure.

Details concerning fMRI data acquisition, preprocessing, single subject and group analyses have been described previously in more detail.^42^ All MR images were analyzed using Statistical Parametric Mapping (SPM5; www.fil.ion.ucl.ac.uk) implemented in MATLAB 6.5 (Mathworks Inc., Sherborn, MA). Axial functional images (n=505, EPI, matrix 64 x 64, 30 slices interleaved, FOV = 230. voxel size = 3.6 x 3.6 x 3.8 mm, TE = 30 ms, TR = 2 s), covering the whole brain and positioned parallel to the intercomissural line (AC-PC) as well as a 3D structural data set (MPRAGE, matrix 128 x 112; 88 slices; FOV = 256; voxel size = 2 x 2 x 2 mm; TE = 3.93 ms; TR = 1100 ms; flip angle = 9) were recorded. Temporal and spatial alignment and normalization into standard stereotactic space (2 x 2 x 2 mm) was performed. Acquisition and extinction phases were split into 1^st^ and 2^nd^ halves to assess time-dependent processes of fear conditioning and extinction training.^43^ Realignment parameters of each subject were considered to account for movement artifacts. Blood oxygen level-dependent (BOLD) responses for each event type (CS+ paired, CS+ unpaired, CS-, US) and phase (F, 1^st^ half A, 2^nd^ half A, 1^st^ half E, 2^nd^ half E) were modeled by the canonical hemodynamic response function within the general linear model.

*Behavioral avoidance test*

The behavioral avoidance test (BAT) procedure was conducted in three phases. Patients were first instructed to sit in front of an open test chamber (75 × 120 × 190 cm) for 10 min (anticipation phase). Next, patients were locked in the dark chamber and instructed to stay as long as possible for a maximum time of 10 min (exposure phase). Terminating exposure was always possible. Subsequently, patients again sat in front of the opened chamber (recovery phase). Upon completion of each BAT phase, participants were instructed to rate the intensity of experienced anxiety during the last period on a visual analogue scale from 1 to 10 and asked, whether they have experienced a panic attack. The tolerated duration of exposure was obtained as an index of behavioral fear response. An electrocardiogram (ECG) was continuously monitored during the whole procedure.
